# Supplementary material for: A Critical View of the Application of the APEX Software (Aqueous Photochemistry of Environmentally-Occurring Xenobiotics) to Predict Photoreaction Kinetics in Surface Freshwaters
Source: Molecules. 2019 Dec 18;25(1):9. doi: 10.3390/molecules25010009 (PMC7017383; doi:10.3390/molecules25010009)
Supplement: Supplementary file 1 [file molecules-25-00009-s001.zip › 3.2.4_gcc-4.4.0.zip!/3.2.4_gcc-4.4.0/3.2.4_gcc-4.4.0/doc/octave/html/interpreter/Matrices-and-Arrays-in-Oct_002dFiles.html]

Matrices and Arrays in Oct-Files - Untitled


Next: Character Strings in Oct-Files,
Previous: Getting Started with Oct-Files,
Up: Oct-Files


---

#### A.1.2 Matrices and Arrays in Oct-Files

Octave supports a number of different array and matrix classes, the
majority of which are based on the Array class. The exception is the
sparse matrix types discussed separately below. There are three basic
matrix types

`Matrix`: A double precision matrix class defined in dMatrix.h, `ComplexMatrix`: A complex matrix class defined in CMatrix.h, and `BoolMatrix`: A boolean matrix class defined in boolMatrix.h.

These are the basic two-dimensional matrix types of octave. In
additional there are a number of multi-dimensional array types, these
being

`NDArray`: A double precision array class defined in dNDArray.h `ComplexNDarray`: A complex array class defined in CNDArray.h `boolNDArray`: A boolean array class defined in boolNDArray.h `int8NDArray` `int16NDArray` `int32NDArray` `int64NDArray`: 8, 16, 32 and 64-bit signed array classes defined in int8NDArray.h, int16NDArray.h, etc. `uint8NDArray` `uint16NDArray` `uint32NDArray` `uint64NDArray`: 8, 16, 32 and 64-bit unsigned array classes defined in uint8NDArray.h, uint16NDArray.h, etc.

There are several basic means of constructing matrices of
multi-dimensional arrays. Considering the `Matrix` type as an
example

- We can create an empty matrix or array with the empty constructor. For
  example

  ```
            Matrix a;
  ```

  This can be used on all matrix and array types- Define the dimensions of the matrix or array with a dim\_vector. For
    example

    ```
              dim_vector dv (2);
              dv(0) = 2; dv(1) = 2;
              Matrix a (dv);
    ```

    This can be used on all matrix and array types- Define the number of rows and columns in the matrix. For example

      ```
                Matrix a (2, 2)
      ```

      However, this constructor can only be used with the matrix types.

These types all share a number of basic methods and operators, a
selection of which include

— Method: T& **operator ()** (octave\_idx\_type)  
— Method: T& **elem** (octave\_idx\_type)  
> The `()` operator or `elem` method allow the values of the
> matrix or array to be read or set. These can take a single argument,
> which is of type `octave_idx_type`, that is the index into the matrix or
> array. Additionally, the matrix type allows two argument versions of the
> `()` operator and elem method, giving the row and column index of the
> value to obtain or set.

Note that these functions do significant error checking and so in some
circumstances the user might prefer to access the data of the array or
matrix directly through the fortran\_vec method discussed below.

— Method: octave\_idx\_type **nelem** (void) const  
> The total number of elements in the matrix or array.

— Method: size\_t **byte\_size** (void) const  
> The number of bytes used to store the matrix or array.

— Method: dim\_vector **dims** (void) const  
> The dimensions of the matrix or array in value of type dim\_vector.

— Method: void **resize** (const dim\_vector&)  
> A method taking either an argument of type `dim_vector`, or in the
> case of a matrix two arguments of type `octave_idx_type` defining
> the number of rows and columns in the matrix.

— Method: T\* **fortran\_vec** (void)  
> This method returns a pointer to the underlying data of the matrix or a
> array so that it can be manipulated directly, either within Octave or by
> an external library.

Operators such an `+`, `-`, or `*` can be used on the
majority of the above types. In addition there are a number of methods
that are of interest only for matrices such as `transpose`,
`hermitian`, `solve`, etc.

The typical way to extract a matrix or array from the input arguments of
`DEFUN_DLD` function is as follows

```
```
     #include <octave/oct.h>
     
     DEFUN_DLD (addtwomatrices, args, , "Add A to B")
     {
       int nargin = args.length ();
       if (nargin != 2)
         print_usage ();
       else
         {
           NDArray A = args(0).array_value ();
           NDArray B = args(1).array_value ();
           if (! error_state)
             return octave_value (A + B);
         }
       return octave_value_list ();
     }
```
```

To avoid segmentation faults causing Octave to abort, this function
explicitly checks that there are sufficient arguments available before
accessing these arguments. It then obtains two multi-dimensional arrays
of type `NDArray` and adds these together. Note that the array\_value
method is called without using the `is_matrix_type` type, and instead the
error\_state is checked before returning `A + B`. The reason to
prefer this is that the arguments might be a type that is not an
`NDArray`, but it would make sense to convert it to one. The
`array_value` method allows this conversion to be performed
transparently if possible, and sets `error_state` if it is not.

`A + B`, operating on two `NDArray`'s returns an
`NDArray`, which is cast to an `octave_value` on the return
from the function. An example of the use of this demonstration function
is

```
     addtwomatrices (ones (2, 2), ones (2, 2))
           =>  2  2
               2  2
```

A list of the basic `Matrix` and `Array` types, the methods to
extract these from an `octave_value` and the associated header is
listed below.

|  |  |  |
| --- | --- | --- |
| `RowVector` | `row_vector_value` | dRowVector.h |
| `ComplexRowVector` | `complex_row_vector_value` | CRowVector.h |
| `ColumnVector` | `column_vector_value` | dColVector.h |
| `ComplexColumnVector` | `complex_column_vector_value` | CColVector.h |
| `Matrix` | `matrix_value` | dMatrix.h |
| `ComplexMatrix` | `complex_matrix_value` | CMatrix.h |
| `boolMatrix` | `bool_matrix_value` | boolMatrix.h |
| `charMatrix` | `char_matrix_value` | chMatrix.h |
| `NDArray` | `array_value` | dNDArray.h |
| `ComplexNDArray` | `complex_array_value` | CNDArray.h |
| `boolNDArray` | `bool_array_value` | boolNDArray.h |
| `charNDArray` | `char_array_value` | charNDArray.h |
| `int8NDArray` | `int8_array_value` | int8NDArray.h |
| `int16NDArray` | `int16_array_value` | int16NDArray.h |
| `int32NDArray` | `int32_array_value` | int32NDArray.h |
| `int64NDArray` | `int64_array_value` | int64NDArray.h |
| `uint8NDArray` | `uint8_array_value` | uint8NDArray.h |
| `uint16NDArray` | `uint16_array_value` | uint16NDArray.h |
| `uint32NDArray` | `uint32_array_value` | uint32NDArray.h |
| `uint64NDArray` | `uint64_array_value` | uint64NDArray.h |
